# Better Targeting, Better Efficiency for Wide-Scale Neuronal Transduction with the Synapsin Promoter and AAV-PHP.B

**DOI:** 10.3389/fnmol.2016.00116

**Published:** 2016-11-04

**Authors:** Kasey L. Jackson, Robert D. Dayton, Benjamin E. Deverman, Ronald L. Klein

**Affiliations:** ^1^Department of Pharmacology, Toxicology, and Neuroscience, Louisiana State University Health Sciences CenterShreveport, LA, USA; ^2^Division of Biology and Biological Engineering, California Institute of TechnologyPasadena, CA, USA

**Keywords:** adeno-associated virus, amyotrophic lateral sclerosis, gene therapy, gene transfer, promoter, synapsin promoter, targeting, TDP-43

## Abstract

Widespread genetic modification of cells in the central nervous system (CNS) with a viral vector has become possible and increasingly more efficient. We previously applied an AAV9 vector with the cytomegalovirus/chicken beta-actin (CBA) hybrid promoter and achieved wide-scale CNS transduction in neonatal and adult rats. However, this method transduces a variety of tissues in addition to the CNS. Thus we studied intravenous AAV9 gene transfer with a synapsin promoter to better target the neurons. We noted in systematic comparisons that the synapsin promoter drives lower level expression than does the CBA promoter. The engineered adeno-associated virus (AAV)-PHP.B serotype was compared with AAV9, and AAV-PHP.B did enhance the efficiency of expression. Combining the synapsin promoter with AAV-PHP.B could therefore be advantageous in terms of combining two refinements of targeting and efficiency. Wide-scale expression was used to model a disease with widespread pathology. Vectors encoding the amyotrophic lateral sclerosis (ALS)-related protein transactive response DNA-binding protein, 43 kDa (TDP-43) with the synapsin promoter and AAV-PHP.B were used for efficient CNS-targeted TDP-43 expression. Intracerebroventricular injections were also explored to limit TDP-43 expression to the CNS. The neuron-selective promoter and the AAV-PHP.B enhanced gene transfer and ALS disease modeling in adult rats.

## Introduction

Adeno-associated virus (AAV) is one of the most widely used systems for gene transfer to the central nervous system (CNS), for example in optogenetics, cre-lox targeting, and CRISPR gene editing. Scientists wishing to genetically modify the brain with AAV often ask: (1) which AAV serotype should I use in my gene transfer experiments; (2) which promoter/expression cassette should I be using; and (3) which route of vector administration gives me the transduction pattern with the greatest disease relevance for my gene of interest? Many of these governing parameters of AAV gene transfer have been extensively studied with focal, intraparenchymal injections. However, few of these comparative studies have been made for wide-scale CNS transduction where neurons are transduced throughout the nervous system. Wide-scale CNS transduction is relevant for modeling and treating CNS diseases with widespread CNS pathology, so optimization of wide-scale gene transfer is important for these goals. This study attempted to improve upon previous work with AAV9 and the hybrid cytomegalovirus/chicken beta-actin (CBA, also known as the CAG promoter) promoter in terms of neuronal targeting and gene transfer efficiency. If possible, then these refinements could be applied to disease modeling and gene therapy.

If neuronal targeting is the goal, then an appropriate cell-type specific promoter is necessary. Synapsin is considered to be a neuron-specific protein (DeGennaro et al., 1983), so its neuron-specific expression pattern could potentially be harnessed to express transgenes in a neuron-specific manner. A minimal human synapsin promoter has been used in adenoviral and AAV vectors for focal injections (Kügler et al., 2003a,b; Shevtsova et al., 2005). An AAV capsid that can reach the CNS after peripheral administration, such as AAV9 (Foust et al., 2009; Wang et al., 2010; Miyake et al., 2011) or other natural AAV serotypes (Miyake et al., 2011; Snyder et al., 2011; Samaranch et al., 2013; Yang et al., 2014; Jackson et al., 2015b) is advantageous for a relatively non-invasive administration that yields wide-scale expression. Now there are several engineered capsids with increased neuronal transduction efficiency (Choudhury et al., 2016a,b; Deverman et al., 2016). Here we tested AAV-PHP.B described in Deverman et al. (2016) in rats for the first time in order to achieve greater gene transfer efficiency and potentially for improved neuronal targeting as well.

The goal of the study was to achieve more efficient CNS transduction in a more targeted manner than our previous studies using AAV9 in rats for the purpose of disease modeling. Transactive response DNA-binding protein, 43 kDa (TDP-43) is an RNA binding protein that is associated with amyotrophic lateral sclerosis (ALS): TDP-43 mutations can cause ALS (Gitcho et al., 2008), and the vast majority of ALS patients harbor TDP-43 neuropathology in post-mortem studies (Mackenzie et al., 2007). We have used the CBA promoter to express TDP-43 before, but here we attempted to better delimit the TDP-43 expression to neurons using the synapsin promoter and intracerebroventricular injections. We studied synapsin promoter-TDP-43 vectors and intracerebroventricular injections in order to address whether the motor paralysis that we see using the CBA promoter is due to TDP-43 expression in the CNS. We also studied AAV-PHP.B TDP-43 vectors since the greater efficiency could lower vector doses needed, which could decrease side effects in the animal and save costs in terms of the amount of vector production that is required. Furthermore, it is better to model an adult onset disease such as ALS in an adult onset animal model. Because adult CNS transduction requires larger amounts of vector relative to transduction of neonates, more efficient vectors will facilitate work in disease-relevant adult subjects. We hope these encouraging results with the synapsin promoter and AAV-PHP.B will help investigators in their design of wide-scale gene transfer studies.

## Materials and Methods

### DNA and Viruses

cDNA for green fluorescent protein (GFP) or human wild-type TDP-43 were incorporated into an expression cassette plasmid including the AAV2 terminal repeats, the CBA promoter, (1.8 kb), the woodchuck hepatitis virus post-transcriptional regulatory element (WPRE), and the bovine growth hormone polyadenylation sequence (Klein et al., 2002). The strong CBA promoter was first incorporated into an AAV vector by Xu et al. (2001). The term CBA promoter is synonymous with the CAG promoter (Niwa et al., 1991). A plasmid for a minimal human synapsin promoter (485 bp) driving expression of yellow fluorescent protein (YFP) was provided by K. Deisseroth, Stanford University. This plasmid also contains the WPRE and human growth hormone polyadenylation sequence. Two different fluorophores were used in this study, GFP and the GFP variant, YFP. YFP should be at least as bright or a brighter fluorophore than GFP (Ormö et al., 1996; Yang et al., 1996). The difference in brightness between the two fluorophores had no bearing on the results particularly since the tissues were immuno-labeled with a GFP antibody that recognizes both proteins. cDNA for human wild-type TDP-43 (gene accession number NM_007375) was also incorporated into the synapsin promoter cassette in place of YFP.

DNAs were packaged into recombinant AAV9 or AAV-PHP.B by described methods (Klein et al., 2008b). Helper and AAV9 capsid plasmids used to generate AAV9 were from the University of Pennsylvania (Gao et al., 2004). AAV-PHP.B capsid plasmids were from the California Institute of Technology (Deverman et al., 2016). Viral stocks were sterilized using a Millipore Millex-GV syringe filter, aliquoted and stored frozen. Viral genome copies were titered using dot-blot assay, and equal titer doses were obtained by diluting stocks in lactated Ringer’s solution (Baxter Healthcare).

### Neonatal Studies

Litters of Sprague-Dawley rats (Envigo) were injected on post-natal day one. A total of 25 neonatal rats were used including both male and female subjects. Animals received 100 μl of virus diluted in lactated Ringer’s solution administered via a 30 g needle into the facial vein, as previously described (Wang et al., 2010). The paws of the animals were tattooed (Spaulding Color, Voorheesville, NY, USA) for identification, and the pups were weaned at 3 weeks of age. The vector doses for neonates are expressed as total vector genomes (vg) injected. The neonatal rats usually weighed 6 g, so the per kg dose is 167 times higher than the total vg injected. Eleven neonatal rats were injected with AAV9 synapsin promoter-YFP at a dose of 4 × 10^12^ vg. Five animals were administered AAV9 CBA-GFP at a dose of 1.9 × 10^12^ vg, and four additional rats were administered AAV-PHP.B CBA-GFP at an equivalent dose of 1.9 × 10^12^ vg.

### Adult Studies

Adult female Sprague-Dawley rats (approximately 6 weeks of age weighing 130 g, Envigo) were used for intravenous administrations. A total of 21 subjects were used. For tail vein injections, animals received 200 μl of virus diluted in lactated Ringer’s solution administered via a 30 g needle into the lateral tail vein. Intravenous delivery was also performed via retro-orbital injections in a subset of animals. For retro-orbital injections, animals received 100 μl of diluted virus administered via a 30 g needle into the capillary bed behind the eye. We were interested in this method to find an easier or more reliable method than tail vein injections. The retro-orbital injections yielded consistent results, but overall we did not notice any obvious, clear-cut advantage compared to the tail vein method in adult rats. Four adult rats were administered AAV9 CBA-GFP at a dose of 1 × 10^13^ vg/kg and one at a higher dose of 7.5 × 10^13^ vg/kg. Four rats were administered AAV9 synapsin promoter-YFP at a dose of 1 × 10^13^ vg/kg and one rat at a higher dose of 7.5 × 10^13^ vg/kg. Three rats were administered AAV-PHP.B synapsin promoter-YFP at a dose of 3.8 × 10^13^ vg/kg.

One adult female Sprague-Dawley rat (225 g) was stereotaxically administered AAV into the substantia nigra (coordinates: 5.3 mm posterior to bregma, 2.1 mm lateral, 7.6 mm ventral). One side was injected with AAV9 CBA-GFP and the other side with AAV9 synapsin promoter-YFP at matching doses (3 × 10^9^ vg). Two adult female Sprague-Dawley rats (approximately 225 g) were stereotaxically administered AAV9 into the lateral ventricle (coordinates: 0.93 mm posterior to bregma, 1.6 mm lateral, 3.5 mm ventral).

### Disease Modeling With TDP-43

To demonstrate the relevance of the GFP results for disease modeling, we administered a subset of rats with TDP-43 vectors. When AAV9 encoding TDP-43 driven by the CBA promoter is administered intravenously to either neonatal (Wang et al., 2010) or adult (Jackson et al., 2015b) rats, a progressive paresis and paralysis of the limbs develops, which may also involve mortality depending on the vector dose used (Jackson et al., 2015b). We have studied rats from 2 to 24 weeks after AAV9 TDP-43 gene transfer (Dayton et al., 2013). TDP-43 is known to induce paralysis, and at a high vector dose, mortality in rats, so we purposefully used small numbers of subjects to determine the presence of the disease state on a yes-or-no basis. Three neonatal rats were administered AAV9 synapsin promoter-TDP-43 at a dose of 4 × 10^12^ vg. Two neonatal rats were administered AAV-PHP.B-TDP-43 at a dose of 1.6 × 10^12^ vg. Three adult rats were administered AAV9 synapsin promoter-TDP-43 at a dose of 3 × 10^13^ vg/kg, and three rats were administered AAV-PHP.B synapsin promoter-TDP-43 at an equivalent dose of 3 × 10^13^ vg/kg. These rats were evaluated for motor dysfunction and mortality. All animal research conducted was approved by the Animal Care and Use Committee at Louisiana State University Health Sciences Center at Shreveport.

### Analysis of Motor Function

Animals were evaluated for motor function via rotarod and escape reflex testing. Rotarod testing was performed on a Rotarod (Rota-rod/RS, Letica Scientific Instruments, Barcelona, Spain) that was accelerating from 4 to 40 rpm over 2 min. The amount of time the rat could remain walking on the rotarod before falling was measured three times and averaged. The escape reflex was evaluated by briefly lifting the rat from the tail. A normal escape reflex is the extension of forelimbs and hindlimbs. Clenching of the fore- or hindlimbs during this test is indicative of a lesion in the motor pathway. Motor deficits for the animals administered TDP-43 were noted if the averaged rotarod scores were less than 30 s or if limb clenching was demonstrated on three consecutive trials.

### Immunohistochemistry

Animals were anesthetized with a cocktail of ketamine (100 mg/ml, Fort Dodge Animal Health, Fort Dodge, IA, USA), xylazine (20 mg/ml, Butler, Columbus, OH, USA), and acepromazine (10 mg/ml, Boerhinger Ingelheim, St. Joseph, MO, USA) in a 3:3:1 fluid ratio. Animals were administered 2 ml/kg of the cocktail intramuscularly before perfusion. The animals were transcardially perfused with phosphate buffered saline followed by cold 4% paraformaldehyde in phosphate-buffered saline. Tissues were removed and immersed in 4% paraformaldehyde overnight at 4°C. Tissues were cryopreserved in 30% sucrose. Fifty μm sections were cut on a sliding microtome with a freezing stage (Leica Biosystems, Buffalo Grove, IL, USA). The primary antibodies were rabbit anti-GFP (Invitrogen, 1:500), mouse anti-GFP (Invitrogen, 1:250), which efficiently label both GFP and YFP, rabbit anti-glial fibrillary acidic protein (GFAP; Chemicon, 1:1000) which labels astrocytes, mouse anti-CD11B (Chemicon, 1:500) which labels microglia, and mouse anti-NeuN (Chemicon, 1:1000) which labels neurons. The secondary antibodies were Alexa Fluor 488 and Alexa Fluor 594 (Invitrogen) at a concentration of 1:300. DAPI (Sigma, St. Louis, MO, USA) was used as a counterstain.

### Analysis of Transgene Expression in The Cerebellum

After immunofluorescent staining for GFP, three evenly spaced sections of the cerebellar vermis for each animal were photomicrographed using a 2.5× lens and converted to grayscale. The fluorescent area was quantified using Scion Image as previously described (Jackson et al., 2015b), and the three sections per animal were averaged. We confirmed that non-transduced tissues had only negligible background readings by analyzing cerebellum that was not transduced with AAV but stained for GFP.

## Results

### Promoter Studies

#### Transgene Expression Pattern of Synapsin Promoter-AAV9

**Figure 1:**
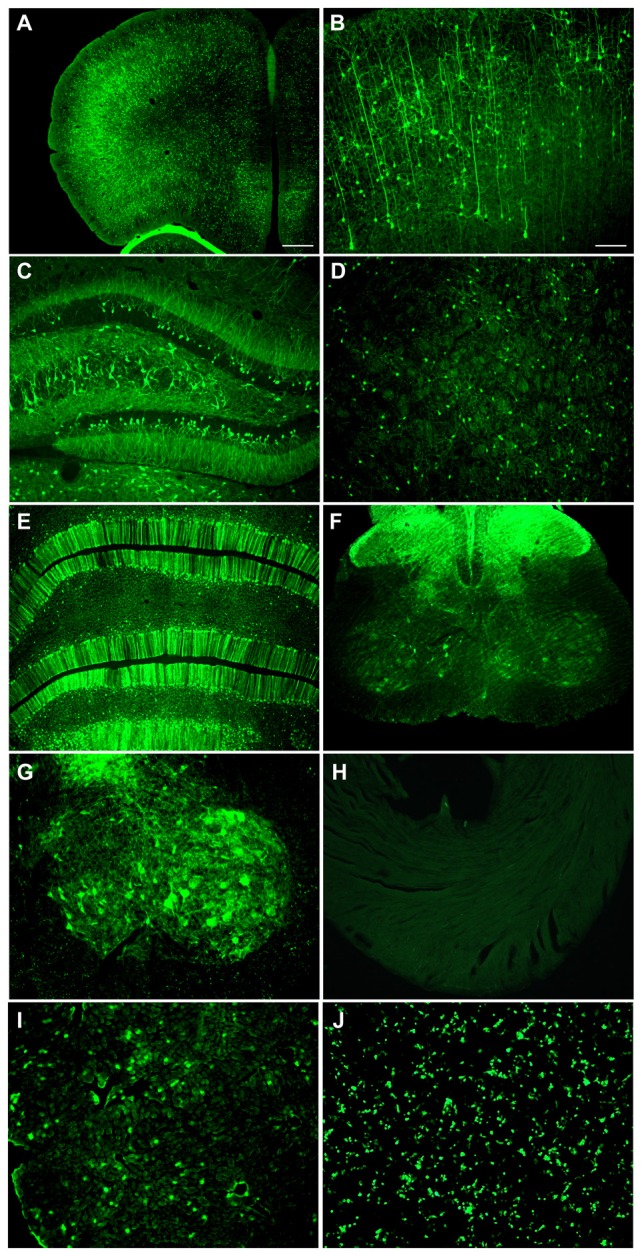
**Highly efficient wide-scale central nervous system (CNS) transgene expression in the rat using the synapsin promoter with avoidance of heart, but not liver expression.** Synapsin promoter-AAV9 was administered intravenously to a neonatal rat and the tissue was analyzed 4 weeks later. **(A,B)** Expression in cortical neurons. **(C)** Hippocampus. **(D)** Striatum. **(E)** Cerebellum. **(F,G)** Spinal cord. **(H)** The heart had trace to no expression. **(I)** The synapsin promoter-AAV9 did result in expression in the liver. **(J)** This synapsin promoter DNA construct also drives expression in non-neuronal cells (HEK 293T) after transfection. Green fluorescent protein (GFP) immunostaining in **(A–I)** Native fluorescence in **(J)** Bar in **(A)** = 268 μm, same magnification in **(E,F,H–J)** Bar in **(B)** = 134 μm, same magnification in **(C,D,G–I)**.

**Figure 2:**
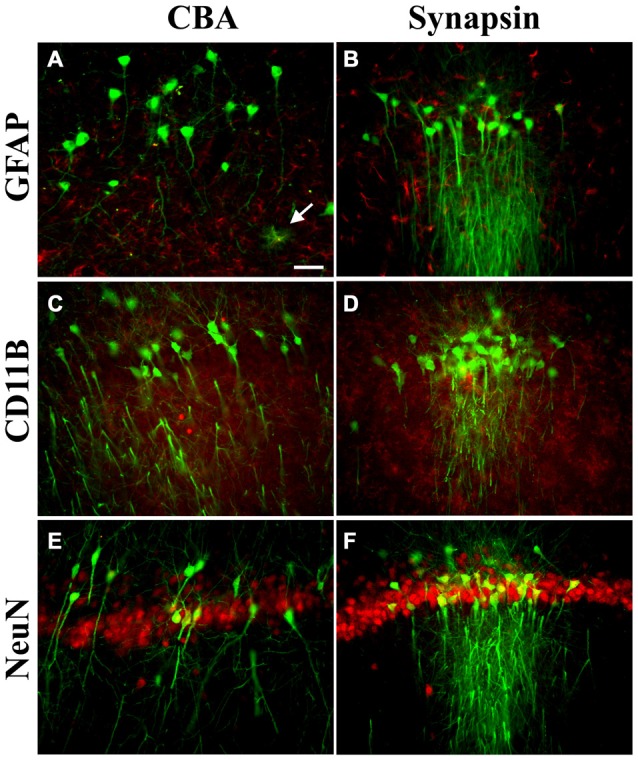
**Transgene expression profiles in astroglia, microglia, and neurons in the CNS.** Pictures are mergers of GFP immunofluorescence in green and cell type specific markers in red, from the hippocampus. **(A,B)** For astroglia (glial fibrillary acidic protein, GFAP), there were occasional examples of GFP-positive cells with the cytomegalovirus/chicken beta-actin hybrid (CBA) promoter (arrow) but GFP-positive astroglia were not present using the synapsin promoter. **(C,D)** For microglia (CD11B), the adeno-associated virus (AAV) transgene did not co-localize with the microglial marker with either promoter. **(E,F)** Most of the cells expressing the transgene in the CNS were co-labeled with the neuronal marker NeuN with either the CBA or synapsin promoter. AAV-PHP.B was used for both promoters and the expression interval was 4 weeks after intravenous administration. Bar in **(A)** = 42 μm, same magnification in **(A–F)**.

A neonatal rat receiving AAV9 synapsin promoter-YFP intravenously at a dose of 4 × 10^12^ vg (6.7 × 10^14^ vg/kg) was evaluated at 4 weeks for CNS expression. Robust, wide-scale expression in the CNS was achieved with efficient labeling of neurons in the frontal cortex, forebrain, cerebellum and spinal cord among other regions throughout the CNS (Figures 1A–G). In the CNS, the transduced cells appeared exclusively neuronal and did not include cells with glial morphologies. The YFP expressed in transduced cells did not co-localize with GFAP, an astrocyte marker, or CD11B, a microglia marker (Figure 2). By comparison, the CBA promoter drives expression in occasional astroglia, though mostly expresses in neurons in the CNS (Figure 2). Outside of the CNS, with the synapsin promoter, the cardiomyocytes were largely spared from transgene expression (Figure 1H), a clear success of this promoter strategy as we were attempting a more restricted pattern compared to the CBA promoter, which efficiently expresses in cardiomyocytes (Wang et al., 2010). However, synapsin promoter driven YFP was observed in the liver at 4 weeks (Figure 1I), so the recombinant human synapsin promoter should therefore be referred to as neuron-selective, not neuron-specific in the context of a recombinant AAV9 vector. Along these lines, during the vector packaging stages, the synapsin promoter expressed in the non-neuronal HEK 293T cells (Figure 1J). Comparing on a qualitative basis with the heart and liver expression conferred by the CBA promoter in rats (Wang et al., 2010), expression in the heart appears to be nearly completely silenced with the synapsin promoter and partially muted in the liver.

#### Comparison of the Synapsin Promoter with the CBA Promoter

**Figure 3:**
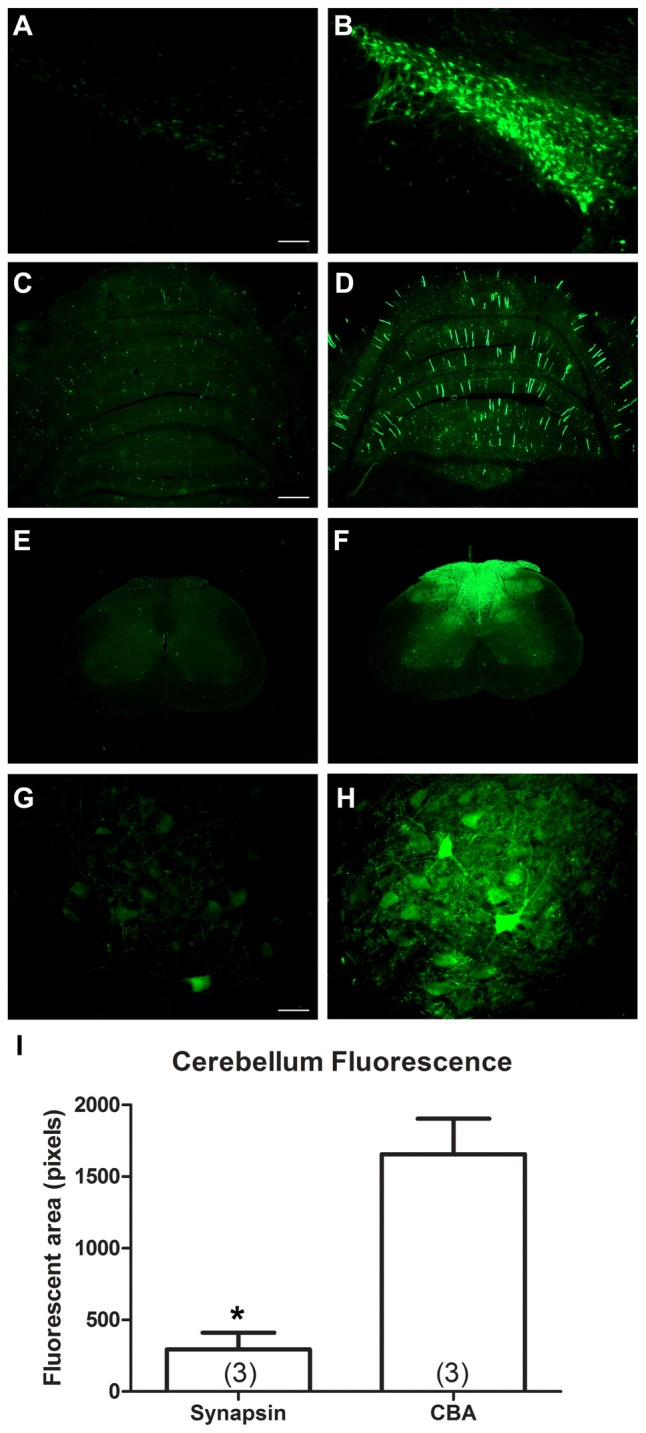
**In comparisons of the synapsin promoter and the CBA promoter, the CBA promoter clearly drives stronger expression.** One rat received a bilateral stereotaxic injection into the substantia nigra. One side was injected with a synapsin promoter-AAV9 and the other with a CBA promoter-AAV9 under equal conditions. **(A,B)** The expression from the synapsin promoter-AAV9, while present, was fainter than the CBA promoter-AAV9 at equal camera exposures. **(C–H)** Similarly, after retro-orbital injections of AAV9 in adult rats under equal conditions, the expression from the synapsin promoter-AAV9 appeared fainter than the CBA promoter-AAV9, cerebellum in **(C,D)** spinal cord in **(E–H)**. **(I)** The GFP-positive area of the cerebellum was greater in the CBA promoter-AAV9 group (*t*-test, *p* < 0.01, *n* = 3/promoter group). GFP immunostaining in **(C–H).** Bar in **(A)** = 134 μm, same magnification in **(B)**. Bar in **(C)** = 536 μm, same magnification in **(D–F)**. Bar in **(G)** = 67 μm, same magnification in **(H)**. *Indicated *p* < 0.01.

The two promoters, synapsin and CBA, were evaluated for CNS expression levels. One Sprague-Dawley rat was administered AAV9 CBA promoter-GFP on one side of the brain and on the other side, AAV9 synapsin promoter-YFP, each at an equal vector dose of 3 × 10^9^ vg. After 3 weeks, the substantia nigra was analyzed for fluorescence, which clearly supported that the CBA promoter drives stronger expression than the synapsin promoter under equal conditions (Figures 3A,B). We then compared the CBA and synapsin promoters in adult rats on a statistical basis, using intravenous gene delivery by the retro-orbital injection method. Each of the two promoter constructs was administered at an equal dose of 1 × 10^13^ vg/kg. The CBA promoter group produced greater expression in the cerebellum compared to the synapsin promoter group by 5.6-fold (Figures 3C–I; *t*-test, *n* = 3/promoter group, *p* < 0.01, expression interval of 4 weeks).

#### Attenuation of Long-Term Expression with the Synapsin Promoter

**Figure 4:**
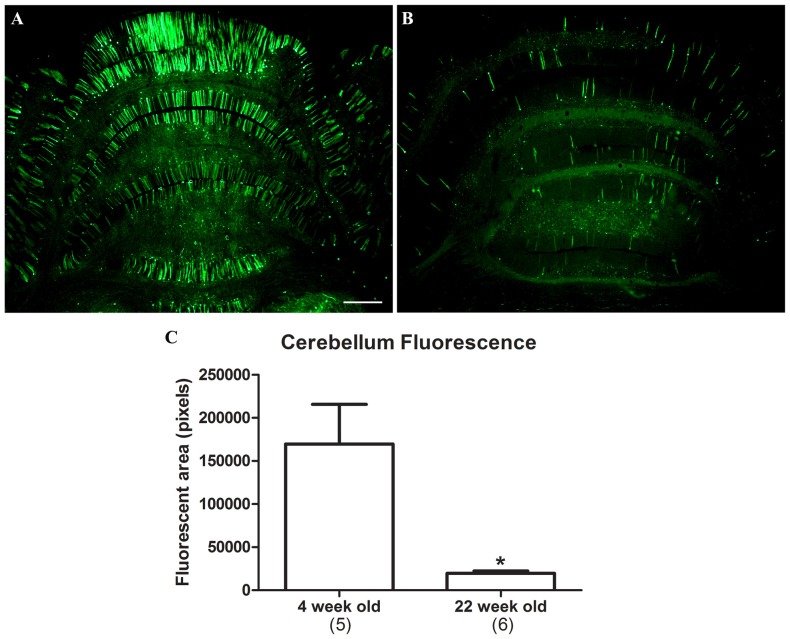
**Attenuation of synapsin promoter-driven expression at 22 weeks post gene transfer.** Neonatal rats were administered synapsin promoter-AAV9 intravenously and the tissues were analyzed at time points of either 4 **(A)** or 22 **(B)** weeks. **(C)** The area of the cerebellum positive for GFP was compared. Lower level expression was found at the later time point (*t*-test, *p* < 0.01, *n* = 5–6 /per interval). GFP immunostaining in **(A,B)**. Bar in **(A)** = 536 μm, same magnification in **(B)**. *Indicates *p* < 0.01.

The CBA promoter is known to confer high expression levels on a long-term basis, for example 1 year in rats in Klein et al. (2002) and 8 months in non-human primates in Jackson et al. (2015a). To determine if expression driven by the synapsin promoter remained stable over time, 11 neonatal rats were administered AAV9 synapsin promoter-YFP at a dose of 4 × 10^12^ vg (6.7 × 10^14^ vg/kg). Five of the rats were evaluated at 4 weeks, and the remaining six were evaluated at 22 weeks. Transduction of the cerebellum was evaluated, which clearly demonstrated attenuation of synapsin promoter driven expression at the longer interval (Figure 4; *t*-test, *n* = 5–6/time point, *p* < 0.01).

### Studies With The AAV-PHP.B Capsid

#### CBA Promoter-AAV-PHP.B, Comparison with AAV9 in Neonates

**Figure 5:**
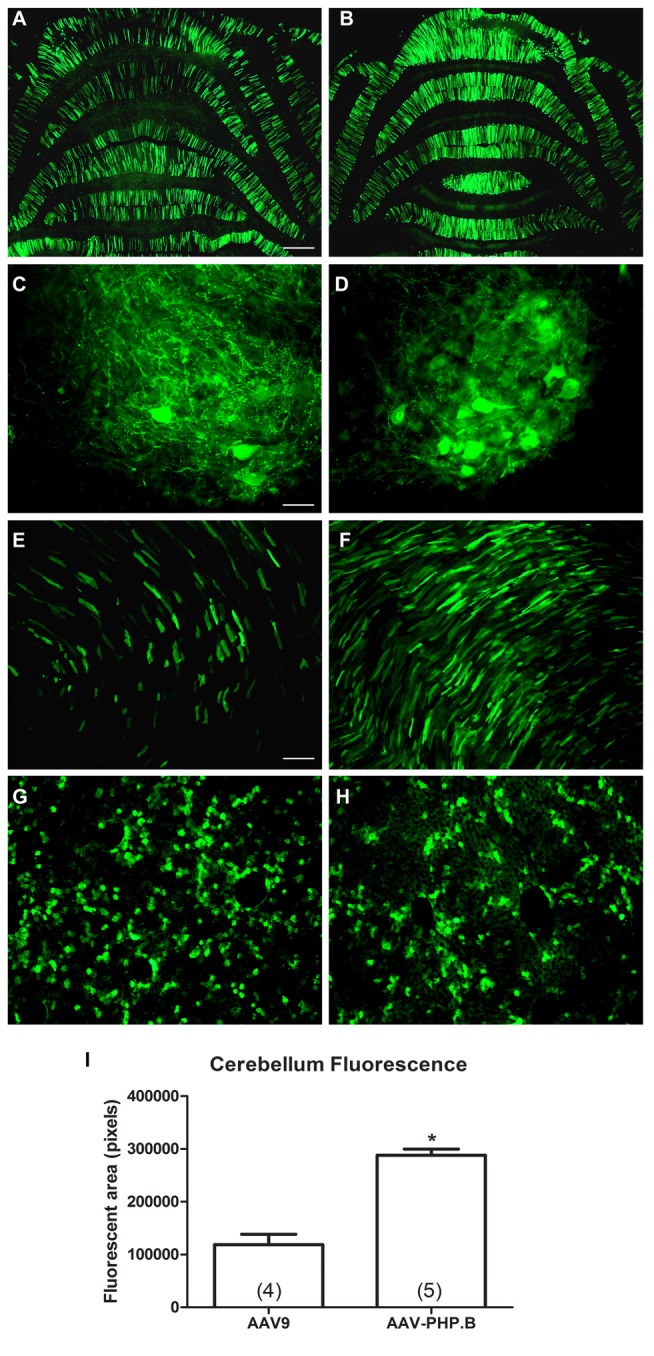
**AAV-PHP.B provides increased transduction efficiency in the CNS relative to AAV9.** Neonatal rats were injected intravenously with the CBA promoter construct packaged into either AAV9 or AAV-PHP.B under equal conditions, with a 4 week expression interval. **(A,B)** There was stronger GFP expression in the cerebella of rats administered AAV-PHP.B. **(C–F)** The same pattern was found in the spinal cord **(C,D)** and the heart **(E,F)**. **(G,H)** Interestingly, AAV-PHP.B did not appear to similarly increase expression in liver, which is consistent with improved neuronal targeting. **(I)** GFP-positive area in the cerebellum (*t*-test, *p* < 0.001, *n* = 4–5/per AAV capsid serotype group). GFP immunostaining in **(A–H)**. Bar in **(A)** = 536 μm, same magnification in **(B)**. Bar in **(C)** = 67 μm, same magnification in **(D)**. Bar in **(E)** = 134 μm, same magnification in **(F–H)**. *Indicates *p* < 0.001.

AAV-PHP.B was reported to be 40-fold more efficient than AAV9 for neuronal transduction in mice by Deverman et al. (2016). Here, we made a comparison in rats using the same CBA promoter expression cassette in AAV9 or AAV-PHP.B. Four neonatal animals were administered AAV-PHP.B CBA-GFP and five neonatal animals with AAV9 CBA-GFP at an equal vector dose of 1.9 × 10^12^ vg (3.2 × 10^14^ vg/kg). Expression was evaluated 4 weeks later. The transduction of the cerebellum was increased by 2.4-fold in the AAV-PHP.B group compared to the AAV9 group (Figure 5; *t*-test, *n* = 4–5/capsid serotype group, *p* < 0.001). The lesser degree of fold-increase observed in rats compared to mice (Deverman et al., 2016) could be due to making this comparison closer to the vector dose saturation point in neonates. Since large doses of highly efficient, high titer AAV9 vectors are needed for wide-scale studies in adult subjects, the increased transduction efficiency of AAV-PHP.B should be able to lower this demand and save on the amount of vector production needed. Though not quantified, there was a visible trend of reduced hepatic expression using AAV-PHP.B compared to AAV9 along with the increased neuronal expression. These results are therefore consistent with the hypothesis that AAV-PHP.B is advantageous for neuronal targeting as well as greater efficiency.

#### Synapsin Promoter-AAV-PHP.B in Adults

**Figure 6:**
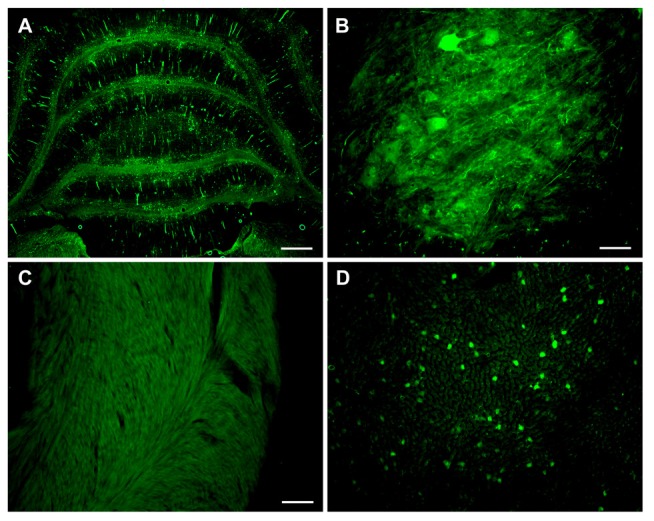
**Combining advantages for targeting and efficiency in a synapsin promoter-AAV-PHP.B vector.** Neonatal rats were administered synapsin promoter-AAV-PHP.B intravenously, with an expression interval of 4 weeks. **(A)** Expression in the cerebellum. **(B)** Spinal cord. **(C)** The heart was blank for expression, as expected. **(D)** Importantly, the liver showed a relatively low level expression compared to previous CBA promoter-AAV9 vectors. GFP immunostaining in **(A–D)**. Bar in **(A)** = 536 μm. Bar in **(B)** = 67 μm. Bar in **(C)** = 134 μm, same magnification in **(D)**.

Based on the increased transduction observed with AAV-PHP.B in neonates, we administered three adult animals with AAV-PHP.B synapsin promoter-YFP at a dose of 3.8 × 10^13^ vg/kg, and the tissues were analyzed 4 weeks later. Neuronal expression was achieved in the cerebellum and spinal cord (Figures 6A,B). There appeared to be no expression in the heart (Figure 6C) and some expression in the liver (Figure 6D), at a lower level than would be expected using the CBA promoter. AAV-PHP.B should therefore be advantageous to boost the expression of a weaker promoter while retaining the promoter tissue specificity.

### Intracerebroventricular Route of Administration

**Figure 7:**
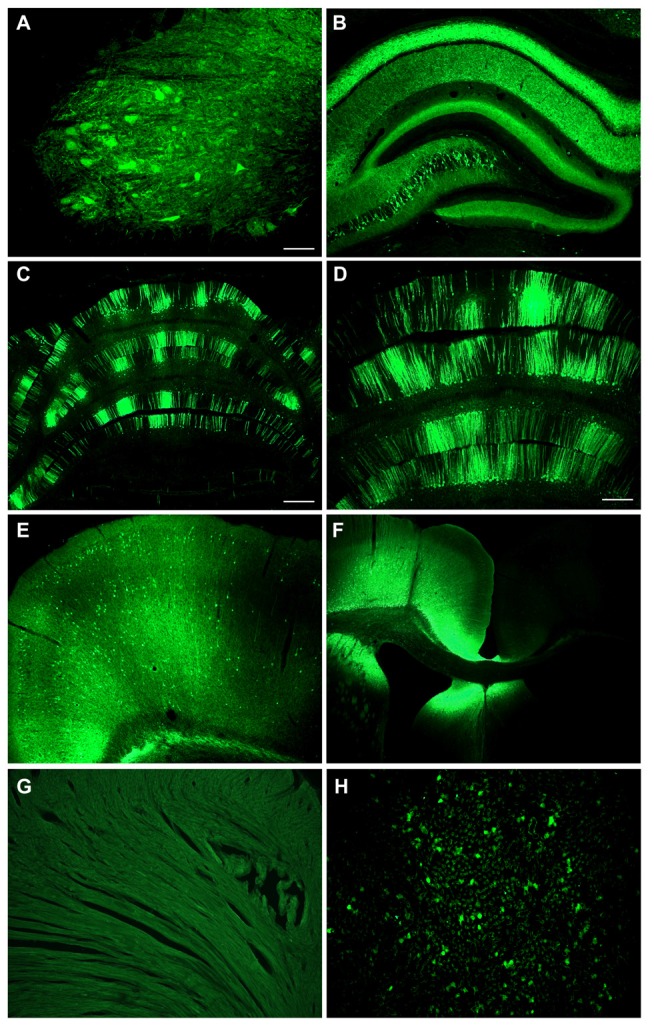
**Intracerebroventricular administration produces far reaching expression in the CNS with low level expression in liver.** An adult rat was injected with CBA promoter AAV9 into the lateral ventricle. There was a widespread expression throughout the CNS including the spinal cord in the lumbar region **(A)**, the hippocampus **(B)**, the cerebellum **(C,D)**, and the cerebral cortex on the contralateral side **(E).** **(F)** There was extremely strong expression along the needle track into the ventricle. **(G)** No expression was seen in the heart. **(H)** There was some expression in the liver. GFP immunostaining in **(A–H)**. Bar in **(A)** = 134 μm, same magnification in **(B,H)**. Bar in **(C)** = 536 μm, same magnification in **(F)**. Bar in **(D)** = 268 μm, same magnification in **(E,G)**.

We also conducted adult intracerebroventricular administrations to improve CNS transduction and limit peripheral transduction. Intra-ventricular AAV injections in mice have been shown to be advantageous for widespread gene transfer in the CNS and gene therapy (Lo et al., 1999; Passini and Wolfe, 2001; Li and Daly, 2002; Passini et al., 2003), whereas intraparenchymal AAV injections in the brain produce more focal expression (Klein et al., 1998, 2002, 2006, 2008a). One rat was administered AAV9 CBA promoter-GFP in the lateral ventricle at a dose of 3.8 × 10^12^ vg/kg, and tissues were analyzed 6 weeks later. There was a wide ranging spread of the GFP expression in the hippocampus, cerebellum and spinal cord (Figure 7). In the hippocampus of this sample, pyramidal neurons in CA3/CA4 were transduced, while the CA1 region showed strong GFP expression in the neuropil though not in pyramidal neuron perikarya. However, a potential caveat of this approach was the very high degree of expression in the cortex along the injection track (Figure 7F), since a large vector dose was used. The cerebrospinal fluid can exit the CNS into the venous system, so peripheral organ transduction was examined. There was little transduction of cardiomyocytes (Figure 7G), but GFP expression in the liver was clearly evident after intracerebroventricular injections (Figure 7H).

### TDP-43-Induced Phenotypes using the Synapsin Promoter, AAV-PHP.B and Intracerebroventricular Delivery

We know that AAV9 wide-scale gene transfer is more efficient in neonates relative to adults (Jackson et al., 2015b). When AAV9 synapsin promoter-TDP-43 was administered to a small group of three neonates, mortality resulted within 2–3 weeks. A relatively high vector dose was used (4 × 10^12^ vg or 6.7 × 10^14^ vg/kg) which would also induce mortality if the stronger CBA promoter was used (Wang et al., 2010). However, in a small group of three adult subjects, we did not observe the typical, progressive motor deficits and paralysis for up to 6 weeks after administering the AAV9 synapsin promoter-TDP-43 at a vector dose of 3 × 10^13^ vg/kg, a dose that is sufficient to induce the disease state using the CBA promoter in adults (Jackson et al., 2015b).

AAV-PHP.B CBA promoter-TDP-43 was noticeably stronger than the AAV9 counterpart in neonates causing severe limb dysfunction and mortality rapidly, by 10 days (1.6 × 10^12^ vg or 2.7 × 10^14^ vg/kg, *n* = 2). In adults, in contrast to AAV9, AAV-PHP.B synapsin promoter-TDP-43 did result in the characteristic paresis/paralysis of the limbs by 2 weeks post-injection (3 × 10^13^ vg/kg, *n* = 3). Both the hindlimbs and the forelimbs were affected in rats in the AAV-PHP.B group during the escape reflex, but no such deficits were noticed in the AAV9 rats. In rotarod testing at 6 weeks post-injection in adults, there was a difference in fall latency between the two AAV serotype groups. The time to fall in the AAV9 synapsin promoter-TDP-43 group was 80.6 ± 10.3 s (unimpaired) and 22.6 ± 13.5 s in the AAV-PHP.B synapsin promoter-TDP-43 group (*p* < 0.05, *t-test*, *n* = 3/group). These results are summarized in Table 1. Interestingly, in one rat administered AAV9 CBA promoter-TDP-43 into the lateral ventricle at a dose of 3.8 × 10^12^ vg/kg, the motor deficits, limb paralysis, and overall disease state manifested within 2 weeks. Importantly, an almost 10-fold lower vector dose was used than what we would use for an intravenous vector dose to induce the disease state with this vector (Jackson et al., 2015b).

**Table 1:** **Outcomes of transactive response DNA-binding protein, 43 kDa (TDP-43)-induced phenotypes in small groups of rats**.

Route	AAV	Promoter	Dose	*N*	Outcome
Neonatal i.v.	AAV9	CBA	2 × 10^12^ vg	1	Fatal by 2 weeks (Dayton et al., 2013)
Neonatal i.v.	AAV9	Synapsin	4 × 10^12^ vg	3	Fatal by 2–3 weeks
Neonatal i.v.	AAV-PHP.B	CBA	1.6 × 10^12^ vg	2	Fatal by 10 days, abnormal limb posture
Adult i.v.	AAV9	CBA	3 × 10^13^ vg/kg	3	Impaired motor function, fatal by 5 weeks post-injection (Jackson et al., 2015b)
Adult i.v.	AAV9	Synapsin	3 × 10^13^ vg/kg	3	No symptoms up to 6 weeks post-injection
Adult i.v.	AAV-PHP.B	Synapsin	3 × 10^13^ vg/kg	3	Impaired motor function by 2 weeks post-injection, *p* < 0.05 vs. AAV9 synapsin
promoter-TDP-43 on rotarod at 6 weeks
Adult i.c.v	AAV9	CBA	3.8 × 10^12^ vg/kg	1	Impaired motor function by 2 weeks post-injection

## Discussion

Better neuronal targeting of wide-scale gene transfer in rats was achieved using the synapsin promoter. Better efficiency was achieved using the AAV-PHP.B capsid. It will be interesting to combine these two elements since the improved capsid can make up for the low promoter strength of this tissue-specific promoter while retaining promoter specificity. This is the first example of gene transfer with AAV-PHP.B in rats. Rats are particularly advantageous compared to mice because of their larger size, because their physiological parameters are closer to human, and because there are specific behavioral, pharmacological, and toxicological assays designed for rats. Furthermore, an increasing amount transgenic rat strains are becoming available.

Within the CNS, the synapsin promoter-AAV9 appeared to avoid glial cells. The CBA promoter-AAV9 mostly expressed in neurons and in some sporadic astroglia. While the synapsin promoter was clearly not as strong as the CBA promoter, successful neuronal targeting was achieved. In contrast to the CBA promoter, the synapsin promoter avoided expression in cardiomyocytes and appeared to generate less expression in the liver. Though not absolutely neuron-specific, the synapsin promoter strategy was successful to mitigate and minimize the peripheral expression to a substantial extent. Neuron-selective, but not neuron-specific expression was also reported by Huda et al. (2014) when they applied a synapsin promoter-AAV9 vector intravenously to mice. The lack of complete neuron specificity of the synapsin promoter may be due to the short recombinant promoter sequence. On the other hand, the AAV2 inverted terminal repeats are known to possess weak promoter activity (Flotte et al., 1993; Haberman et al., 2000) which could be responsible for the non-neuronal expression. Additionally, some studies have found synapsin protein and mRNA in the liver (Bustos et al., 2001) suggesting that endogenous synapsin expression may not be neuron-specific. In any case, we were able to achieve quite robust wide-scale transgene expression with the synapsin promoter-AAV9 in rats for the first time. The efficiency of the synapsin promoter-driven expression throughout the CNS shown here is unprecedented. Critically important, and in contrast to previous results with the CBA promoter, we report reduced synapsin promoter-driven expression in the long-term (5–6 months). More work will be needed to confirm this effect and perhaps determine if the synapsin promoter is subject to silencing by DNA methylation, for example Prösch et al. (1996). The lowered expression over time with the synapsin promoter would probably not affect short-term studies on the order of 1 month, but could be critical to consider in long-term studies including neurodegenerative disease modeling. Furthermore, the widespread, diffuse expression pattern after intravenous injections may be more sensitive to detect reduced expression over time than in stereotaxic injections which introduce more genome copies per transduced cell. The relatively low to moderate expression conferred by the synapsin promoter may be advantageous for achieving physiologically relevant expression levels, and clearly the synapsin promoter is advantageous for neuronal targeting with the wide-scale approach.

We found that AAV-PHP.B yielded higher transduction efficiency for neurons in rats than AAV9, corroborating the results in mice in Deverman et al. (2016). We exploited this critical advantage by coupling the synapsin promoter with AAV-PHP.B which enabled a phenotype including both forelimb and hindlimb motor paralysis when expressing TDP-43. We were unable to observe a phenotype with AAV9 synapsin promoter-TDP-43 in adult rats, but this hurdle was overcome using the more efficient AAV-PHP.B, underscoring the utility of the improved engineered AAVs.

By observing the TDP-43-induced phenotype with the synapsin promoter in neonates with AAV9 and in neonates and adults with AAV-PHP.B, we are more confident that the paralysis is mediated by TDP-43 expression in neurons. We were somewhat surprised the intracerebroventricular injection resulted in such far reaching expression in the CNS, although similar findings have been reported with intra-cerebrospinal fluid injections in mice, pigs, and monkeys (Snyder et al., 2011; Federici et al., 2012; Samaranch et al., 2013; Donsante et al., 2016). We found a mosaic, sporadic labeling of clusters of Purkinje neurons in the cerebellum after intracerebroventricular administration, consistent with other studies (Hinderer et al., 2014; Donsante et al., 2016), which may reflect the flow of the cerebrospinal fluid. In contrast, the intravenous delivery produces more evenly distributed transduction in the cerebellum by comparison. As previously discussed (Gholizadeh et al., 2013; Samaranch et al., 2013; Donsante et al., 2016), the intraventricular route of administration is advantageous for wide-scale CNS transduction because it better limits the vector to the CNS and requires lower vector doses relative to intravenous delivery. We also found some degree of hepatic transduction after intracerebroventricular administration in rats as seen before in mice and monkeys (Samaranch et al., 2013; Donsante et al., 2016). We can assume that using the synapsin promoter and AAV-PHP.B would better avoid this low level expression in the liver after intracerebroventricular administration. The intracerebroventricular delivery method was advantageous for better targeting TDP-43 expression to the CNS and clearly sufficient to induce the characteristic phenotype. Thus, the paralysis should be mediated by TDP-43 expression in brain and spinal cord neurons. One caveat of this study is the low sample size used for the stereotaxic injection of CBA and synapsin promoter vectors and for the intracerebroventricular gene transfer. However, we compared the CBA and synapsin promoters on a statistical basis after i.v. administration and found a similar pattern to the focal injection, that the CBA promoter is clearly stronger. For the intracerebroventricular injections it was clear that wide-scale expression can be achieved in rats and that this method permits the characteristic TDP-43 induced paralysis. We believe that the combination of the synapsin promoter and AAV-PHP.B will be advantageous to investigators asking which configuration to use in their studies, given the improved targeting and efficiency. Though preliminary, we realized the benefits of an intracerebroventricular route of administration, while an intravenous delivery is less invasive. While not absolutely neuron-specific, the synapsin promoter did produce a highly selective expression pattern which supports that greater and greater pinpoint targeting can be achieved even after peripheral administration of vector.

We conclude that the combination of the synapsin promoter with AAV-PHP.B is advantageous for neuronal targeting and efficient expression in adults after peripheral, wide-scale intravenous delivery. We demonstrated that this combination was necessary for observing behavioral motor deficits induced by TDP-43 in adults, with neuron-selective expression. If this design worked for achieving our goal with TDP-43 here, then synapsin promoter AAV-PHP.B vectors will probably also permit new basic and clinical neuroscience approaches that were not possible before.

## Author Contributions

RLK and BED were involved in the conception and design of the work and the interpretation of the data. KLJ and RDD were involved in the acquisition, analysis and interpretation of the data. KLJ, RDD, BED and RLK: were involved in drafting and revision of the manuscript, approval of the final version, and agreed to be held responsible for the work.

## Funding

This work was funded by the ALS Association and Karyopharm Therapeutics, Inc.

## Conflict of Interest Statement

BED is listed as an inventor on a patent application related to AAV-PHP.B. KLJ, RDD and RLK have no conflicts of interest to disclose.
