# Corrigendum: Better Targeting, Better Efficiency for Wide-Scale Neuronal Transduction with the Synapsin Promoter and AAV-PHP.B

**DOI:** 10.3389/fnmol.2016.00154

**Published:** 2016-12-22

**Authors:** Kasey L. Jackson, Robert D. Dayton, Benjamin E. Deverman, Ronald L. Klein

**Affiliations:** ^1^Department of Pharmacology, Toxicology, and Neuroscience, Louisiana State University Health Sciences CenterShreveport, LA, USA; ^2^Division of Biology and Biological Engineering, California Institute of TechnologyPasadena, CA, USA

**Keywords:** adeno-associated virus, amyotrophic lateral sclerosis, gene therapy, gene transfer, promoter, synapsin promoter, targeting, TDP-43

The following acknowledgments were missing from the original article.

BD was supported by the Hereditary Disease Foundation, the Beckman Institute and the Resource Center for CLARITY, Optogenetics and Vector Engineering, which supports technology development and dissemination at the California Institute of Technology. The AAV-PHP.B plasmid was distributed from the Gradinaru Laboratory at the California Institute of Technology. The authors thank Viviana Gradinaru for her help in providing the AAV-PHP.B plasmid.

The authors apologize for this mistake. This error does not in any way change the scientific conclusions of the article.

## Conflict of interest statement

BED is listed as an inventor on a patent application related to AAV-PHP.B. KLJ, RDD and RLK have no conflicts of interest to disclose.

